# Moving beyond informal action: sustainable energy and the humanitarian response system

**DOI:** 10.1186/s41018-021-00102-x

**Published:** 2021-11-04

**Authors:** Peter James Matthew Thomas, Sarah Rosenberg-Jansen, Aimee Jenks

**Affiliations:** 1 University of Bristol, Senate House, Tyndall Ave, Bristol, BS8 1TH UK; 2 Refugee Studies Centre, University of Oxford, 3 Mansfield Road, Oxford, OX1 3TB UK; 3 United Nations Institute for Training and Research, UNITAR, Palais des Nations, CH-1211 Geneva 10, Switzerland

**Keywords:** Energy access, Energy planning, Refugees, Displacement settings, Humanitarian response, Cluster system

## Abstract

Energy and humanitarian action have long been uneasy bedfellows. In the field, many humanitarian practitioners lack the time or remit to engage with a complex issue such as energy, and the topic to date has received relatively little attention from the private, development and academic sectors. This paper hopes to provide more clarity on energy in forced displacement settings by analysing how energy is interwoven with the humanitarian cluster system. This paper has two aims: (1) to assess existing evidence in the sector and explain the links between energy and each of the humanitarian clusters and (2) to provide recommendations on how humanitarian response efforts can transition from informal action to a comprehensive response on sustainable energy provision. This paper is the first to investigate the role of energy using the cluster system as a framework and contributes to a rapidly evolving field of research and practice on energy in humanitarian contexts. Our analysis demonstrates that energy is not fully integrated within humanitarian programme planning. Further, it highlights pathways for improving humanitarian outcomes enabled by improved energy practices. We identify ten ways clusters can integrate action on energy to support crisis-affected communities.

## Introduction

Humanitarian situations affect approximately 120 million people every year (Clarke and Parris 2019) and currently there are around 70.8 million forcibly displaced people living worldwide (UNHCR 2019a), a figure which has increased significantly in recent years (UNHCR 2011). Displacement situations are seen as a strain on the already stretched resources of developing economies, where an estimated 80% of displaced people live (UNHCR 2020a). Although energy-related issues have appeared on the humanitarian agenda for over 20 years (Bellanca 2014), with energy recognised by the United Nations High Commissioner for Refugees (UNHCR) as “a basic need for everyone” (UNHCR 2015), energy has received relatively little focus overall in humanitarian practice. Existing research has already established that energy has not been systematically considered in response planning and is underfunded by humanitarian agencies (Lahn and Grafham 2015; D’Annunzio et al. 2016; Gerrard 2016; Lehne et al. 2016; Thulstrup and Joshi 2017; Corbyn and Vianello 2018). This is despite recognition that energy is essential for ensuring well-being and livelihoods (Pachauri and Spreng 2004; Nussbaumer et al. 2012; Nerini et al. 2017; Jeuland et al. 2021), as well as an increasing awareness amongst humanitarian practitioners that energy is a key resource for enabling the security of people in vulnerable situations.

Recent initiatives at the international level have drawn attention to the importance of energy and sought to improve coordination in humanitarian contexts. Examples include the working group on Safe Access to Fuel and Energy in Humanitarian Settings established in 2007 to facilitate a more coordinated and timely response to the fuel and energy needs of conflict-affected populations (Bellanca 2014), the Moving Energy Initiative (MEI) programme implemented in 2014–2018 to elevate the importance of humanitarian energy in policy and practice (Chatham House 2019), and the Global Platform for Action on Sustainable Energy in Displacement Settings (GPA) launched in 2018 with the objective of systematically integrating sustainable energy into humanitarian response (UNITAR 2018). At the field and national levels, energy and environment working groups have been established in some countries (for example in Uganda, Jordan and Bangladesh) to coordinate sustainable energy programming. There has also been an increase in the number of humanitarian energy projects including the Humanitarian Engineering and Energy for Displacement project (Coventry University et al. 2019), the Alianza Shire projects (2017), the SET4food project (Barbieri et al. 2018), and the Renewable Energy for Refugees project (Practical Action 2019).

Despite this initial progress, the delivery of sustainable energy solutions remains an under-researched and under-valued topic within humanitarian response (Barbieri et al. 2018). The existing cluster system and humanitarian coordination mechanisms have been slow to integrate sustainable energy planning in practice. For example, a recent survey of Rohingya refugees in Bangladesh found that energy was their most important unmet need (Hopkins and Hetzer 2018). Despite emerging action, existing mechanisms have fallen short of providing regular or sustainable access to clean cooking or modern energy services.

The objective of this paper is to present evidence from the existing literature regarding the role of energy across the humanitarian response system and to critically reflect on the current provision of energy services within it. This is the first paper to investigate energy access and use in humanitarian relief in this way and supports the sector’s collective understanding of the practice of humanitarianism and how issues of protection, technology and governance connect with the energy sector. This paper provides a summary on energy within the humanitarian clusters and the high-level analysis needed to support sector-specific guidance on integrating energy within the humanitarian system. The article then sets out recommendations on how energy can be further embedded within the individual cluster sectors.

The following sections outline our research approach and present an overview of existing energy provision in humanitarian response. The clusters are presented in three sections and analyse sector-specific literature in a similar order to the provision of humanitarian assistance which broadly follows the Universal Declaration of Human Rights (United Nations, 1949), whereby the right to protection is closely followed by an individual’s right to shelter, food, health, education and work. Initially, we explore what type of energy is needed for basic human survival and its intersection with the protection, shelter and settlements and household items, food security and nutrition clusters. Second, sustainable energy provision for essential services and its intersection with the health, WASH and education clusters is dicussed. Third, we explore how renewable energy can be utilised to power humanitarian operations through the intersection with the Camp Coordination and Camp Management (CCCM), logistics and emergency telecoms clusters. Finally, we describe these mechanisms and provide specific measures which could be adopted by the clusters to embed sustainable energy solutions across cluster policies and programming.

## Research approach and methodology

A wide range of topics which discuss energy in humanitarian response including emergencies and protracted crises were reviewed as part of the analysis for this paper. Since there is limited peer-reviewed work on humanitarian energy (Rosenberg-Jansen 2018), non-academic publications have also been reviewed including case studies, project documents and reports.

The review was conducted using the Scopus research database and a set list of keywords developed by the authors. Separate searches of the Journal of International Humanitarian Action and Journal of Humanitarian Engineering were conducted using the same keywords because these were not indexed by Scopus. Searches were limited to Title, Abstract and Keyword, and only English language publications were included, as the majority of the analysis published on this topic is in English and only then occasionally translated into other languages. Grey literature available on key websites such as Energypedia, ReliefWeb, the individual humanitarian cluster websites, the websites of key humanitarian organisations, and unpublished evidence and data available to the authors was also included. The reference lists of texts identified during the literature search were also reviewed and any texts not already identified were evaluated. Over 400 documents were identified to provide the foundation of the analysis. Evidence collected through informal discussions with practitioners is also presented. These discussions build on the experience of the authors in delivering humanitarian energy interventions and conducting academic research at the humanitarian energy nexus. Reflecting on these lessons and experience within this paper brings balanced and critical reflections to this research article to support the evidence presented from academic sources.

The paper uses the Inter-Agency Standing Committee (IASC) Cluster System as a framework for understanding the role energy currently plays in humanitarian response and opportunities for further integration in the future. The cluster system is only activated in non-refugee humanitarian emergencies (UNHCR 2020b), however, it closely mirrors the sectoral working groups implemented by the UNHCR under the Refugee Coordination Model (RCM) in refugee situations (UNHCR 2020b). As a result, it provides a good framing to present a sectoral analysis across the technical and humanitarian intervention areas. Examining the cluster system also enables us to understand how energy connects to issues of humanitarian protection, response programming within aid agencies, and the role energy technologies play within the humanitarian sphere.

The paper is primarily focused on displaced people residing in camps or similar settlements in developing countries, which fall under the mandate of international humanitarian organisations. In these contexts, energy is classed as a service which can be provided alongside other critical areas of aid such as water, food, and shelter. In other displacement contexts, a national government or other agencies might provide alternative methods of support on energy. For example, following an earthquake or flood, governments may support energy services through their national electricity suppliers. Under national government remits, technical energy experts are often in charge of the response and use well-established or national delivery mechanisms. In the humanitarian sector, however, responses are often uncoordinated and rely on individuals with a basic operational knowledge of energy. It is for this reason that we focus on camps or similar settlements to understand the role humanitarian actors and the cluster system plays in delivering energy to displaced people. However, it is recognised that many displaced people live in urban or peri-urban areas, reliant on the local infrastructure, which is outside the scope of this analysis but still requires research.

## Humanitarian response and energy access

In this paper, energy access includes cooking, heating and cooling and the electricity needs of people in displaced settings, including energy use by field-practitioners. It includes access within homes, enterprises, community facilities and humanitarian operations. This paper uses the terms “humanitarian energy” and “energy in displacement settings” interchangeably to cover sector-wide action on energy provision within humanitarian response. The intersection of humanitarian response and energy, informally referred to as humanitarian energy, can be defined as the “institutions, policies, programmes, global initiatives, actions and activities which use a range of sustainable and fossil fuel energy sources in contexts of displacement to meet the energy needs of people in camps and urban settings, self-settled refugees, host communities, and Internally Displaced Peoples (IDPs)” (Rosenberg-Jansen 2020).

**Fig. 1:**
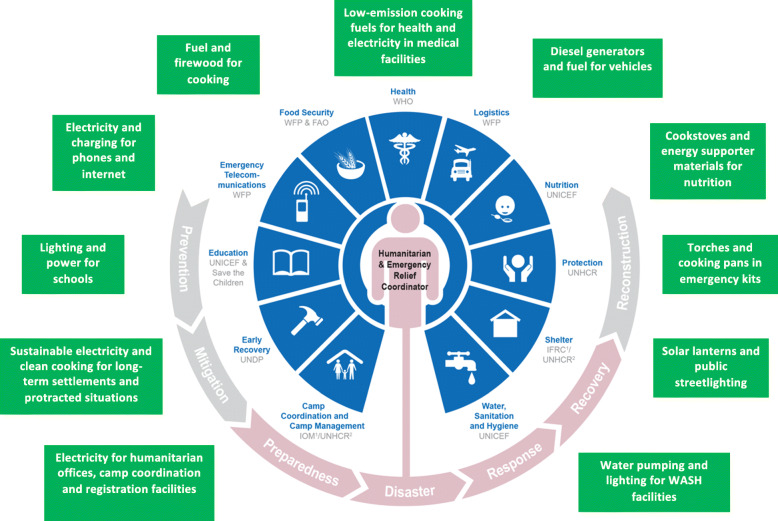
The cluster system with examples of energy uses. Source: Adapted from (OCHA, 2020)

Humanitarian services, including energy responses, have traditionally been provided under the “protect and provide” model often present in emergency response, where camps or settlements are set up (theoretically for the short term) to offer a physically protective space for displaced people. Humanitarian agencies then provide products to meet their basic needs for shelter, food, water and safety. Responses are led by humanitarian aid agencies including the UNHCR, the International Organization for Migration (IOM), the World Food Programme (WFP), the Food and Agriculture Organization (FAO), and the United Nations Office for the Coordination of Humanitarian Affairs (OCHA), alongside Non-Governmental Organisations (NGOs) and humanitarian partners. These organisations coordinate action using a range of processes, including the cluster system, the RCM and working groups on specific issues. Specifically, the cluster system (Fig. 1) coordinates over-arching action across a range of technology and intervention areas, such as water, protection, health and logistics (IASC 2015). Each cluster has a lead agency responsible for coordinating the delivery of humanitarian assistance within that sector.

Figure 1 provides a preliminary overview based on the authors’ experience of the areas where energy interlinks with the cluster system. It also highlights that there is currently no official cluster for energy despite previous attempts to create one (Bellanca 2014; Callaghy 2020). As a result, energy responses are currently only connected informally to the existing cluster system. This is problematic because previous research has established that significant gaps exist in sectors and on specific cross-cutting issues where there is no clearly mandated lead agency (Van Dorp 2009). This means that energy solutions are often not supplied despite increasing recognition that it is essential for improving people’s quality of life and should be viewed as a human right (Bradbrook and Gardam 2006; SE4ALL 2017; Kyte 2019). Where energy has been provided, it has been in a largely ad hoc manner through methods of distributing products and services for free (Bellanca 2014; Lahn and Grafham 2015; Thulstrup and Joshi 2017; Corbyn and Vianello 2018).

One often-cited reason for the lack of governance on energy provision in humanitarian response is that it is not viewed as an essential need during emergencies (UNITAR 2019). However, many international commitments have proposed that humanitarian and development responses must be linked from the early stages of a crisis rather than once a situation becomes protracted (Agenda for Humanity 2016; Idris 2017; UN 2018a; UN 2018b; UNITAR 2018). Delivering long-term solutions is vital because humanitarian situations are increasingly becoming long term (ALNAP 2018), and protracted situations are becoming the “new normal” (Sova 2017; Boodhna et al. 2019). Often the processes and coordination mechanisms which are appropriate for short-term response are not suitable for protracted a situation (Bennett 2015; Sanderson et al. 2015; Knox Clarke and Campbell 2018). Energy is a quintessential example of this. The distribution of energy products in emergencies works in the short term. For example, the provision of solar lanterns and cookstoves helps meet the basic needs of vulnerable populations by preventing starvation and providing basic lighting for safety. However, following the initial phase of an emergency (after around six months)^1^, such distribution mechanisms no longer meet people’s needs. Households need more diverse sources of energy, communities need street-lighting and power for basic public services like health and water pumping, and humanitarian operations need large amounts of electricity to continue their work (UNITAR 2018). Humanitarian aid organisations are not set up to deliver such support and often it is outside their core remits. The sections below consider the evidence on energy within the humanitarian system, presented under each of the clusters to summarise how access to energy is viewed from a cluster perspective.

### Energy for protection and survival

Energy is essential for the delivery of core humanitarian services such as powering registration services and the transportation of people and/or equipment. It is also needed to ensure the basic survival of displaced populations such as for cooking food and heating shelters. This section covers energy provision across the protection, shelter, food security and nutrition clusters. Household items have also been included in the shelter cluster because this is the typical structure implemented under the RCM.

#### Protection

The UNHCR leads the protection cluster which aims to ensure that the rights and dignity of displaced people including legal and physical protection is achieved. Although the UNHCR recently produced an energy strategy (UNHCR 2019b), its protection remit means that most of the assistance provided so far has focused on cooking rather than electricity. Cooking has traditionally been considered a protection issue because when displaced people collect their own fuel, they may face conflicts with local communities, increased risks of being involved in an accident, or become more exposed to situations in which gender-based violence can occur (Hassen 2006; Musse 2012; Spangaro et al. 2013; Gunning 2014; Gianvenuti et al. 2015; Thulstrup and Henry 2015). Most of the evidence regarding the nexus between energy and protection in the existing literature regards firewood collection for cooking. However, safety in dark public spaces and feeling secure within camp and non-camp environments is also a major protection challenge where energy plays an important role.

Multiple studies connect firewood collection and violence against displaced people, including examples in Uganda, Ethiopia, Chad, Darfur, South Sudan, Namibia, Nepal, Kenya and the Democratic Republic of Congo (Médecins Sans Frontières 2005; Patrick 2006; Lyytinen 2009; Danish Refugee Council 2012; Gunning 2014; Global Alliance for Clean Cookstoves 2015; Gerrard 2016). The literature demonstrates that the burden and risks of collecting firewood falls overwhelmingly on women and girls (Lyytinen 2009; Booker et al. 2012; WRC 2014; Thulstrup and Joshi 2017; Listo 2018), exposing them to greater risks. However, it is important to note that most gender-based violence is inter-partner and inter-familial violence that happens within or close to homes (Duvvury et al. 2004; Kishor and Johnson 2004; World Health Organization 2005; Grafham 2020), rather than ‘stranger’ violence which happens during firewood collection. There are some examples of inter-partner firewood violence in the literature such as Mulumba (2011) who found that the time women spent away from their homes collecting firewood had a negative effect on their domestic relations. While protection narratives primarily focus on women and girls, there is also some evidence of violence against men occurring, particularly men who earn an income producing charcoal (Thulstrup and Joshi 2017; Bermudez et al. 2018; Hastie et al. 2019).

To address the protection challenge associated with firewood collection, humanitarian agencies have provided fuel and improved cookstoves (which reduce the amount of fuel required) to displaced people. However, most of the literature demonstrates that there is little evidence that providing basic fuel or improved clean cookstoves meets protection needs (CASA Consulting 2001; Abdelnour 2015; Listo 2018). For example, in Darfur, despite receiving improved cookstoves, firewood distribution was still insufficient to meet displaced people’s needs, and women were forced to collect firewood (Patrick 2006). This has created a perception amongst NGOs that improved cookstoves have little or no impact on protection (Langol and Wolf 2005). Furthermore, a recent review on the role that cookstoves and fuels have on preventing gender-based violence found that there was a lack of compelling evidence as to whether and how cookstove and fuel projects reduced the risk and overall frequency of gender-based violence (Global Alliance for Clean Cookstoves 2016).

Although many protection debates have tended to focus on violence, displaced communities face several other protection-based issues regarding energy. Displaced people are sometimes confined to camps, unable to legally move outside of these spaces to access energy. This presents several legal challenges that align with the remit of the protection cluster such as refoulement. There are examples in Bangladesh, Djibouti and Ethiopia where refugees have been arrested for illegal fuelwood collection, and in Tanzania, there are incidences of refoulement after refugees left camps for various reasons including firewood collection (Lyytinen 2009). Where movement is allowed, tensions and conflict between host and refugee communities can build due to dependence on local natural resources such as firewood for cooking. Furthermore, according to the MEI, “the fact that firewood collection outside camps is illegal in many countries further encourages exploitation of the vulnerable and under-reporting of assaults” (Lahn and Grafham 2015).

Several reports link lighting and the protection remits in humanitarian contexts (McKinsey 2010; Merieau and Egziabher 2012; Munz and Rasoul 2013; IOM 2014). For example, one of the reasons women fear using communal kitchens and sanitation facilities is a lack of lighting (Gunning 2014; Hastie et al. 2019). Lighting around clinics and hospitals also increases the perception of public safety and acceptability of health services (Gunning 2014). Increased perception of safety is not just related to sexual and gender-based violence. For example, in Kenya and Nepal, it has been reported that lights helped people spot snakes and scorpions at night (FaIDA 2013). As a result, street lighting is often a high priority for camp residents (Corbyn and Vianello 2018) and the UNHCR has provided solar street lights to camps such as in Bangladesh, which had a positive impact on the overall security situation (Fuentes et al. 2018). However, in the past, poorly designed programmes have meant that streetlights have had a high failure rate, with theft and poor maintenance common issues (Corbyn and Vianello 2018). Furthermore, lighting is not a panacea for protection and can present its own challenges. For example, despite receiving solar lamps, women in Haiti still perceived their camps as unsafe (Dynes et al. 2014) and Gunning (2014) found that in some circumstances such as in the Democratic Republic of Congo, people did not use their lights because they were worried it would enable rebel groups to identify their location.

This review has so far focused on how energy is currently connected to the remit of the protection cluster. However, in line with others (e.g. Harrell-bond and Chambers 1986; Steets et al. 2016; UNHCR 2016; Betts and Collier 2018), our analysis suggests that the protection remit on energy could be expanded. For example, household expenditure on energy should be considered more carefully because displaced people spend between 15 and 31% of their income and resources on energy (Lahn and Grafham 2015; Corbyn and Vianello 2018). Such high levels of expenditure represent a huge burden on displaced people and could be considered part of the protection remit of humanitarian agencies. Therefore, the protection cluster has an important role to play in both advocating for the energy needs of displaced people and finding ways to reduce the financial burdens placed on refugees and displaced people who fund their own energy access.

In conclusion, the connection between energy and protection is complex, with evidence demonstrating that the insufficient provision of cooking fuel and lighting can exacerbate protection issues such as sexual and gender-based violence (SGBV) and refoulement. Most research has focused on reducing firewood collection as a way of preventing SGBV. However, there is increasing evidence that this approach does not address the root causes of SGBV (Abdelnour 2015). Improving access to light has faced similar challenges and the impact of projects to date has been limited. However, there have been no projects that fully address all household and community lighting needs.

#### Shelter and household items

The shelter cluster is co-chaired by the International Federation of Red Cross and Red Crescent Societies, the body responsible for leading in disaster situations, and the UNHCR who lead in conflict situations. The cluster is responsible for overseeing the identification and planning of humanitarian settlements and the coordination of shelter actors to ensure displaced people have access to safe, dignified and appropriate shelter. In many contexts the shelter cluster is also responsible for the supply of household items, often referred to as non-food items (NFIs).

To date, only a proportion of household energy needs have been met. For example, in some contexts, solar lanterns and clean cookstoves have been provided (IKEA Foundation 2014; WakaWaka 2017; WakaWaka 2018). However, the shelter cluster is increasingly seen as an appropriate place to integrate a more comprehensive household energy response. For example, energy has been successfully integrated into the shelter cluster in recent emergency responses in Bangladesh and Venezuela. In Bangladesh, the shelter cluster set minimum standards for household lighting and Liquefied Petroleum Gas (LPG) stoves (Shelter/NFI Cluster 2019), and in Venezuela, the cluster distributed over 1,129 solar lamps (OCHA 2020). However, this is not the case in all situations, and although the sphere standards recognise the importance of energy, they make limited recommendations on minimum standards (Sphere Association 2018). A failure to provide clear guidance and the setting of minimum energy access targets in the sphere standards leaves humanitarian practitioners without a benchmark or common guidance to work towards. However, this is beginning to change. At the 2019 Global Refugee Forum, the UNHCR made a commitment that all refugee and host community households will have Tier 2 electricity access by 2030 (UNHCR 2019c).

The construction of shelters using wood, as both a construction material in shelters and to fire bricks for shelter construction, adds further pressure on depleting local wood sources (Birendra and Nagata 2006; Thulstrup and Joshi 2017). As a result, some governments, such as Rwanda, have responded by restricting the use of wood for shelter construction (Lyytinen 2009). Furthermore, a review conducted by the Women’s Refugee Committee recommended providing material for shelter construction to avoid exacerbating environment degradation and tension with host communities (WRC 2011). Humanitarian settlements are typically densely packed and represent a high risk of fire (Atiyeh and Gunn 2017). The reliance that communities have on traditional energy sources (such as open fires, kerosene lanterns and candles) exacerbates this risk (Gunning 2014; Lahn and Grafham 2015), and there are a number of examples of fires in humanitarian camps in the literature (UNHCR 2008; UNHCR 2013; Medecins Sans Frontieres 2020). A systematic review carried out by Kazerooni et al. (2016) found that fires in refugee and displaced persons settlements (excluding urban settings where most displaced people reside) have resulted in at least 487 deaths and 790 burn injuries since 1990. While there is some evidence suggesting that the distribution of solar lamps has decreased the risk of fires (IOM 2014), no comprehensive studies have investigated this. Ensuring the safe design of camp cooking areas can also help to reduce fire risk (WRC 2014). However, a Building Research Establishment Trust analysis of fire risk in humanitarian camps found that while better camp design would reduce the risks of fire spread, it was often not possible to adhere to the standards in terms of the required spacing between shelters (Shipp and Annable 2008).

In many climates, such as Afghanistan, there is a need for heating shelters in winter and cookstoves often double as a room heater during cold weather (Gunning 2014). In countries such as Jordan, organisations have supported displaced families to thermally insulate their homes to reduce the need for heating in winter (Lahn and Grafham 2015; Lahn et al. 2016). There is a close link between the shelter and health cluster here because insufficient heating can be a serious health risk in colder climates (Zabaneh et al. 2008; Lahn and Grafham 2015). There is also a link to the logistics cluster because supplying sufficient fuel to ensure comfortable conditions for occupants in poorly insulated accommodation can create a major logistical challenge (Obyn et al. 2015). In warmer climates such as Jordan, Libya, and Iraq, fans, and air conditioners are needed (IRIN 2015; Dupin 2018). Some newly designed shelters also have insulation to improve thermal comfort in both winter and summer (Better Shelter 2018). However, these “modern” shelters have been criticised as being inappropriate in many contexts (Scott-Smith 2019).

Many authors now suggest that moving to new forms of humanitarian delivery, such as alternative procurement processes and market-based solutions, could improve the effectiveness of cluster action on energy (Oxfam and WFP 2013; Whitehouse 2019). In particular, the impact of the free distribution of household items such as solar lanterns by shelter cluster members has been criticised (Cohen and Patel 2019), as these technologies often do not meet the needs of households and families have no choice concerning the products they receive. This could be enabled by linking energy to cash assistance (IRENA 2019) such as the inclusion of energy in minimum expenditure baskets in Uganda (WFP 2019).

#### Food security and nutrition

Co-chaired by the WFP and the FAO, the food security cluster aims to ensure food availability and utilisation. The nutrition cluster is led by the United Nations Children's Fund (UNICEF) and aims to safeguard and improve nutritional standards. In non-emergency humanitarian situations, food security is typically its own sectoral working group and nutrition is combined with the health group.

Although food is often provided in humanitarian situations, access to fuel and cooking technologies is often overlooked and poorly funded (Thulstrup and Henry 2015; Caniato et al. 2017). As fuel and cook stoves are classified as NFIs, they often fall under the responsibility of the shelter cluster, leading to a disconnect between the food being delivered and the tools used to cook it. This is a significant oversight because in some cases up to 95% of basic foods are not fully digestible without adequate cooking (Gunning 2014). As a result, ensuring that food security and nutrition targets are achieved are challenges inherently linked to the supply of energy in humanitarian contexts.

Households often employ a variety of cooking technologies (D’Annunzio et al. 2016). Three-stone fires and traditional cookstoves are the primary forms of cooking in most settings in sub-Saharan Africa (Galitsky et al. 2005; Gunning 2014). Most households rely on firewood as a result, and estimates suggest 21-91 kg is consumed per person per month depending on the type and quantity of food cooked, stove efficiency and cooking practices (D’Annunzio et al. 2016). Notably, previous research has found people are reluctant to admit to firewood collection (Patrick 2006) and existing estimates may be unreliable. In most cases, the amount of fuel or firewood distributed by aid agencies is typically not enough to meet the needs of displaced communities (Lyytinen 2009). For example, in the Farchana camp in Chad, 7 kg was provided per person per month (Gunning 2014). As a result, households collect firewood from the surrounding environment to meet their needs and, although existing estimates vary depending on the context, this can involve walking up to 15 km and take up to 8 h per trip (Langol and Wolf 2005; Rogers et al. 2013; Global Alliance for Clean Cookstoves 2015). However, previous research has established that there is a tendency to underestimate the amount of time spent collecting firewood (Langol and Wolf 2005). Most studies reviewed here suggested that clean cookstove programmes reduced firewood collection by approximately 50% (Langol and Wolf 2005; Patrick 2006; Lyytinen 2009; Global Alliance for Clean Cookstoves 2015; Lahn and Grafham 2015).

A range of cooking technologies and fuels are available that potentially alleviate the need for firewood completely, including solar powered electric cooking (Batchelor et al. 2018) and LPG (UNHCR 2014; UNHCR 2017; UNHCR 2018a). In situations where alternative fuels and cooking technologies are promoted, implementation however is not without challenges. For example, the cost of alternative fuels can be a barrier for refugee households (Patel and Gross 2019) or a high operating expense for the humanitarian operation, and fuels can also face logistical and political issues even where policies are implemented to promote them (Rogers et al. 2013). In Nepal and Chad, people sold the provided kerosene fuel to purchase firewood instead (ProAct 2012). Furthermore, positive outcomes of clean cookstove interventions have rarely been significant or sustained because clean cookstoves have not been widely adopted and they have not sufficiently replaced traditional cookstoves (Wilson et al. 2016). Solar cookers are a good example of this, as in most cases solar cookers have been ineffective (ProAct 2012). Corbyn and Vianello (2018) found that despite the perceived advantages of solar cookers in sun-rich regions being clear, solar cookers failed for multiple reasons including the inability to cook when it is not sunny, the impact the cookers had on the way food tasted, the inability to store the large cooker in small houses, and because of a lack of training. There is recognition in the literature that cookstove programmes are more complex than many organisations recognise, and require significant planning and expertise to implement well, which is inhibited by short-term budget cycles (USAID 2007).

A lack of fuel and appropriate cooking technologies in humanitarian contexts often causes people to adopt negative coping mechanisms such as undercooking food, reducing the frequency and size of meals, only cooking once a day, choosing between which family members get to eat, skipping meals, selling food for fuel or switching to less nutritious foods that have a shorter cooking time (Langol and Wolf 2005; Lyytinen 2009; Gianvenuti et al. 2015; Muthiah and Aleinikoff 2015; Thulstrup and Joshi 2017; Mendum and Njenga 2018; Sandwell et al. 2020). In situations where firewood collection is not possible, displaced communities need to purchase cooking fuel which reduces the amount of money they have available to spend on food (Caniato et al. 2017).

There is evidence of markets for cooking fuels and cooking technologies in most displaced settings (Rogers et al. 2013). However, innovative delivery and funding models are needed to mitigate the challenges associated with limited access to funding for cooking and nutrition support (Caniato et al. 2017; Patel and Gross 2019). Adopting market-based approaches such as providing vouchers or embedding energy into cash initiatives enables the acquisition of goods in the open market (Vianello 2016; Caniato et al. 2017; IRENA 2019; Patel and Gross 2019). This also enables displaced people to choose the solution most appropriate to their needs and increases their sense of ownership over the solution (Patel and Gross 2019). However, this is potentially difficult to achieve in contexts where “free resources” such as firewood are locally available; displaced people are likely to utilise these and spend vouchers or cash on other needs.

According to Aste et al. (2017), food preservation due to a lack of technologies and access to energy is one of the most neglected pillars of food security in humanitarian contexts. In Jordan, refugees reported that their diets improved after they received access to electricity because they were able to refrigerate food (Dupin 2018). The potential of evaporative cooling technologies which cool through the evaporation of water and are common across sub-Saharan Africa is also often overlooked in humanitarian settings (Cross et al. 2019). Energy can also play an important role in contexts where displaced people are engaged in agricultural activities. For example, solar systems can be used for irrigation and water distribution during food production; to power machines for crop processing, milling and packaging; and to charge mobile phones that can be used for selling products and mobile banking (WFP 2018). Resources such as the Toolbox on Solar-Powered Irrigation Systems and the Powering Agriculture portal can be utilised by humanitarian staff in situations where solar irrigation is possible to support improved livelihoods and food security (Energypedia 2020a; Energypedia 2020b; Energypedia 2020c).

In conclusion, energy for food security and nutrition are inherently interwoven with cooking energy needs because, without fuel or firewood, displaced people struggle to cook edible food. This section has focused therefore on cooking needs, as this is the primary area where food and energy connect. This review finds that much of the literature on food and energy has focused on the free distribution of clean cookstoves, rather than considering how market-based or alternative delivery models may meet the needs of displaced people. It is also worth noting that the majority of projects and research on energy and food has been focused on sub-Saharan Africa and it is not clear to what extent the findings of these can be applied to other displacement settings.

### Energy for essential services and livelihoods

Energy access is needed for essential humanitarian services and to improve the livelihoods of displaced communities. In these sectors, energy is needed to power healthcare facilities, for water pumping to ensure access to clean water, and to support access to electricity in schools and educational facilities.

#### Health

Led by the World Health Organization (WHO), the health cluster aims to relieve suffering and save lives while improving the well-being and dignity of displaced communities. Most energy health-related research has focused on households (Suhlrie et al. 2018) and a nexus between energy, health and food is apparent within the literature. According to Barbieri, Riva and Colombo (2017), this is caused by a lack of technologies for appropriate and safe food utilisation which leads to malnutrition and weak health and enhanced causes of mortality. Comparatively little discussion exists regarding energy provision in health facilities, an important omission considering modern energy access plays a critical role in the capabilities of healthcare facilities (Porcaro et al. 2017). Within the current context of the COVID-19 crisis, energy needs and the health sector are likely to become increasingly pressing: both in terms of the provision of electricity to power healthcare centres and clean cooking solutions to reduce respiratory risks for indoor air pollution (UNITAR 2020).

Electricity is required in healthcare facilities for lighting, laboratory services, vaccine, blood and medicine storage, and equipment sterilisation (Gunning 2014; Porcaro et al. 2017; Suhlrie et al. 2018). It is also important for the delivery of maternal and neonatal healthcare (Adam et al. 2005; Say and Raine 2007) and can help medical facilities attract and retain staff (WHO 2015). In Yemen, fuel shortages have caused several health-related challenges because hospitals and drug manufacturers are unable to operate without electricity (Burki 2016). Furthermore, as part of a systematic review investigating the availability and safety of blood transfusions during humanitarian emergencies, Abdella, Hajjeh and Sibinga (2018) found that a reliable power supply was one of the major challenges associated with maintaining the availability and safety of the blood supply system. While no data could be found for humanitarian contexts, estimates in the development literature suggest that 60% of refrigerators used to store vaccines and medications face unreliable electricity supplies (Gavi Alliance 2012). Ensuring households have access to energy also improves the ability of refugees to store medication themselves (Dupin 2018). Further health-related impacts identified in the literature include a reduction in bicycle accidents as a result of improved street lighting and a reduced risk of contracting a cold and general feeling of well-being associated with the use of washing machines to clean clothes (Dupin 2018). The lack of light and power in camps and urban situations also drives displaced people to deploy high-risk coping strategies such as power theft which risks electrocution (Lahn and Grafham 2015).

Energy and health are also connected through the negative impacts of air pollution and respiratory risks. Displaced communities typically rely on traditional fuels which emit high concentrations of pollutants including carbon monoxide, particulate matter and other organic compounds when burned (Gunning 2014; Barnes 2014; Albadra et al. 2020). These pollutants can cause a range of negative health issues including respiratory problems, headaches, tuberculosis, eye disease, cancers, low birth weight and increased rates of pneumonia (Dherani et al. 2008; Pennise et al. 2009; Pokhrel et al. 2010; Rogers et al. 2013; Gunning 2014; Albadra et al. 2020). Based on WHO data, the MEI estimated that reliance on polluting fuels causes 20,000 premature deaths amongst displaced people per annum (Lahn and Grafham 2015). These negative health impacts disproportionately affect women and young children who spend large amounts of time cooking; such impacts are especially dangerous where cooking takes place inside (Gianvenuti et al. 2015; Thulstrup and Henry 2015). Most of the literature on indoor air pollution focuses on cooking. However, there are other causes of indoor air pollution, including burning kerosene for lighting (Gunning 2014). While clean cookstoves can enable a reduction in indoor air pollution they can still breach WHO guidelines for indoor air pollution (Pennise et al. 2009). The issue is particularly acute for refugee communities. For example, Muthiah and Aleinikoff (2015) found that acute respiratory infection mortality rates were up to seventeen times higher amongst refugee communities in Nepal and up to four times higher in refugee communities in Burundi when compared to their non-displaced peers.

Another health concern when using traditional fuels is the risk of poisoning (Qudaih et al. 2013). For example, there is evidence that children are sometimes poisoned by accidentally consuming kerosene (Lahn and Grafham 2015). This is further exacerbated by the common practice of insecure storage in soft-drink bottles (Lam et al. 2012). No estimates could be found specific to humanitarian contexts. However, according to Tshiamo (2009), the ingestion of kerosene fuel and resultant poisoning is a leading cause of childhood morbidity and mortality in developing countries. In some contexts, such as Ethiopia, there is also a fear amongst refugees of using ethanol fuels because of safety issues (Rogers et al. 2013). Traditional approaches to cooking such as three-stone fires and lighting using hurricane lamps also significantly increase the risk of burns (Peck et al. 2008; Gianvenuti et al. 2015). Displaced communities in Somalia reported that burns were the biggest health hazard encountered with traditional cooking methods (Musse 2012). Again, no humanitarian specific figures could be found but Mills (2016) found that more than 95% of deaths from burns worldwide occur in low- and middle-income countries where most forcibly displaced people reside. There are several other health impacts emerging from using traditional fuels. For example, research conducted by Pieterse and Ismail (2003) found that incidences of diarrhoea increased when firewood was scarce because people cooked food for more than one day, increasing the likelihood of bacteria developing in the food. In some contexts, displaced women also carried firewood loads of 20 kg or more, putting them at risk of dehydration and short- and long-term physical injury (Rogers et al. 2013; Muthiah and Aleinikoff 2015).

In conclusion, while some literature exists on the connections between health and energy in displacement settings, this has focused on two issues: energy provision of health services and the negative health impacts of cooking fuels. Provision of sustainable services for health needs often seems to be focused on the provision of solar or hybrid electricity and energy-efficient appliances for health clinics, and rarely extends to interventions for households. There is also intermittent action on energy and health under the current cluster system and health-focused agencies lack the support needed in this area.

#### Water, sanitation and hygiene

Led by UNICEF, the WASH cluster aims to ensure the equitable and culturally acceptable provision of water sanitation and hygiene services. Energy plays a critical role (Butler et al. 2013) and is needed for water pumping, purifying drinking water and providing clean water to shower and bathe. Warm water is also needed by hospitals and health clinics for sterilising equipment and providing warm baths for newborn babies and mothers who have just given birth.

Supplying water in emergency situations can be both costly and inefficient (UNHCR 2018b). For example, analysis by Fohgrub (2018) established that 60% of the diesel used in the Nyarugusu refugee camp in Tanzania was used for groundwater pumping. Several studies have established that the life cycle costs of solar-powered systems are more cost-effective than generator-powered systems (Odeh et al. 2006; Meah et al. 2008; Runo and Muema 2014; Andreasi Bassi et al. 2018). Replacing diesel-powered water pumps with a solar- or hybrid-powered (diesel and solar) system in camps in South Sudan had an average payback period of approximately 1.4 years (IOM 2017). A global assessment estimated that implementing solar water pumping in all the UNHCR managed camps could save US$ 43 million over a 20-year period (Ossenbrink et al. 2018). Solar-powered systems can replace diesel-powered systems and are a viable option in most camps (Armstrong and Nakafeero 2016; Corbyn and Vianello 2018). According to Kraehenbuehl et al. (2015), solar-powered systems are especially viable where fuel supply is challenging due to logistical or security constraints. However, several solar water pumping projects have encountered issues with the theft of solar panels and the initial capital costs of systems remains a barrier (Runo and Muema 2014). According to Corbyn and Vianello (2018), the most significant barrier to the successful solarisation of water points in the camps is the low solar technical expertise of WASH field teams. However, efforts to overcome these barriers have been made through the development of technical guidance (i.e. UNDP 2019; Energypedia 2020a; Llario and Kiprono 2020).

Evidence also exists in the literature of a nexus between WASH, protection and energy risks. For example, night-time use of WASH facilities is often limited due to a perception of insecurity, particularly amongst women and children, caused in part by a lack of lighting (Merieau and Egziabher 2012; Gunning 2014; Regattieri et al. 2018; Hastie et al. 2019). In Doro camp, South Sudan, the Danish Refugee Council (2012) found that 52% of 131 respondents reported incidents of violence against women at water points. This is recognised in the UNHCR WASH Manual (UNHCR 2018b), which also notes the challenge of men congregating around female WASH facilities if these are the only facilities with lighting.

Managing waste within refugee camps is also highly challenging (Regattieri et al. 2018) and unprocessed waste can have serious impacts on human health (Connolly et al. 2004; Waring and Brown 2005; Garfì et al. 2009; Zakaria et al. 2018). Technologies that process waste and convert it into energy such as biogas digesters could be an effective solution to this challenge (Regattieri et al. 2018; Makhanu and Waswa 2018). In Kakuma, Kenya, faeces were combined with other waste products, including charcoal dust, to create briquettes for heating or cooking (Nyoka et al. 2017). The briquettes burn with much lower indoor concentrations of carbon monoxide compared to traditional charcoal and reduced the need for refugee households to collect firewood (Karahalios et al. 2018). However, the success of these projects has been limited because of a lack of access to finance to initiate and scale projects, along with the challenges associated with competing with donated products and a lack of clarity on private-humanitarian partnerships (Grafham 2020). Although projects are starting to explore the implications of e-waste (i.e. Innovation Norway 2020), what happens to electronic waste such as solar products and batteries when they reach the end of their life has also been neglected (Cross and Murray 2018; Kumar and Turner 2020).

In conclusion, although proven technologies exist such as solar water pumps, the use of renewable energy for water pumping is not yet standard operating practice. Many water pumping systems, especially in refugee camps, still rely on diesel generators and expensive fuels. Considerable cost and efficiency savings could be made by switching water pumping and WASH facilities to sustainable power sources (Grafham and Lahn 2018; Ossenbrink et al. 2018).

#### Education

Co-led by UNICEF and Save the Children, the education cluster aims to ensure a timely, effective and coordinated education response in humanitarian crises. Energy is needed to enable children to study at night and to power school and training facilities, and in some cases, cooking fuels and technologies are needed to cook school meals.

Access to electricity in households, particularly for lighting, is identified in the literature as an enabler of good education delivery. Light enables children to study in the evenings and electricity enables access to learning technologies (Merieau and Egziabher 2012; Gunning 2014; Moss et al. 2014). Refugees often report that better access to electricity improved their children’s ability to study (FaIDA 2013; Dupin 2018). However, this type of feedback is potentially unreliable and other authors have found limited evidence that study time increased (Corbyn and Vianello 2018), or that learning outcomes improved (Furukawa 2014) following improved access to just lighting. Evidence from the development literature also suggests that current research on energy and education is simplistic and fails to properly consider gender, socio-economic status or local economic factors (Kumar 2018). Access to electricity in schools allows classrooms to remain open before or after the sun sets and facilitates the use of connected technologies such as computers, internet access, printers and projectors. For example, in Jordan, the Renewable Energy for Refugees project installed solar systems and energy efficiency upgrades to reduce expenditure following the Syrian refugee crisis (Practical Action and UNHCR 2018). Having a reliable energy source can also help schools attract and retain teaching staff (UNDESA 2014; Welland 2017).

There is also evidence in the literature of an energy-food-education nexus. Schools in Kenyan refugee camps charged students who did not bring firewood for cooking their lunch meal (Gunning 2014). Women and girls also have less time to participate in educational programmes because they need to collect firewood for their households (Lyytinen 2009; FAO 2018). In Uganda, Mulumba (2011) observed girls dropping out of school to look after younger siblings while their mothers collected firewood, and in Ethiopia, children missed school because they had to collect firewood (Tadele and Getaneh 2016). Kumar (2018) also suggests that providing electric lights benefits boys and men more than girls and women. However, no studies investigating this issue in depth could be found in a humanitarian context.

Education can also ensure that users know how to operate modern energy systems correctly and play a role in dispelling myths and improving trust (Scott 2017; Ebers Broughel 2019). For example, evidence from the development literature indicates that households may not be aware of their energy use or the options available to them (Sovacool 2013; Kapoor et al. 2014). Households with higher education are also more likely to switch from dirty fuels (Urpelainen and Yoon 2015; Joshi and Bohara 2017; Baul et al. 2018; Yadav et al. 2019). However, households with higher education levels also tend to be wealthier and have a higher ability to pay for energy as a result. Furthermore, crisis-affected people are more likely to keep and use stoves or alternative fuels if they are educated about the benefits of the product and trained in its proper use and maintenance (Global Alliance for Clean Cookstoves).

In conclusion, much more research needs to be done on the links between energy and education. To date, there has been a focus on schools as a primary physical location, but in the future, the education cluster could consider the wider economic situation and how home and social environments are connected to energy.

### Energy for humanitarian facilities and operations

As well as providing energy for displaced communities, humanitarian agencies and partners also need energy for their own operations, including power for the offices and residences of humanitarian staff, electricity for registration spaces and fuel to support humanitarian logistics. However, reducing emissions from energy use has tended to be seen as impeding the primary objective of delivering humanitarian assistance (Grafham and Lahn 2018). Until recently, sustainable energy for humanitarian operations had not been considered in a substantive or sustainable way (UN Environment 2017; Grafham 2020). However, recognising the need for responsible action, the United Nations has made commitments to achieve climate neutrality (UN Environment 2017), although progress towards this aim has been slow.

#### Camp coordination, logistics and telecoms

The CCCM cluster is led by the IOM in disaster situations and the UNHCR in conflict situations. It aims to ensure that humanitarian assistance in all settings is well coordinated, supports the governance of operations, and ensures the representation of refugees in decision-making processes. WFP leads the logistics which facilitates access to logistics services and provides information management and the telecoms cluster which aims to provide shared communication and connectivity services.

Limited attention has been paid to energy use in humanitarian compounds (Bellanca 2014). Moreover, responsibility for powering camp facilities often falls to humanitarian logisticians who lack knowledge and experience of energy systems which means they typically implement diesel generators which are often over- or underloaded (Bellanca 2014; Lahn and Grafham 2015). In some contexts, administrative offices without power must conduct work using pen and paper or mobile phones (Corbyn and Vianello 2018).

Exacerbated by the often remote location of displaced people, the provision of fuel for generators can be both expensive, costing upwards of US$ 0.60–2.00 per kWh, and energy intensive (Kraehenbuehl et al. 2015; Grafham and Lahn 2018; Mozersky and Kammen 2018). Furthermore, according to Disparte (2007), in many humanitarian organisations, vehicle fleets represent their second largest operating costs after staff. The difference between a well-managed and poorly managed vehicle over a 6-year period can run to over US $35,000 (Herrmann 2006). While many agencies collect data on their energy usage it is not currently reported in a standardised way or separated from other uses such as transport, making the assessment of alternative approaches challenging (Lahn and Grafham 2015; Grafham and Lahn 2018; Gibson 2020). Maintenance provision is also a challenge, and although modern energy systems often include remote monitoring systems, these rely on phone networks that can be unreliable in some humanitarian settings (Lahn and Grafham 2015).

Electricity is also needed for information and communication technologies in humanitarian settings. For example, charging mobile phones and powering radios that allow displaced communities to keep in touch with friends and family and enabling access to important information (Gunning 2014). Access to mobiles can also help support livelihoods and income generation opportunities (Betts et al. 2014; GSMA 2017; Corbyn and Vianello 2018), and can also enable the delivery of telemedicine services (Latifi and Tilley 2014; Porcaro et al. 2017). However, phones require charging, which refugees often express as one of their top energy-related priorities (Sacino 2017; GSMA 2019). Electricity is also required to provide access to internet services (Brown and Mickelson 2018).

It is not clear that consistent or considered collaboration on providing operational energy systems is currently happening. For example, Fuentes et al. (2018) identified a lack of training, funding and cooperation between the different agencies as the main reasons for the failure of solar systems in Algerian refugee camps. There are also examples in humanitarian contexts of energy products being unused or re-sold (Boodhna et al. 2019). As a result, local capacity development is an important factor in successfully delivering energy interventions (Beck and Martinot 2004; Radulovic 2005; Urmee and Harries 2009; Brooks and Urmee 2014). However, appropriate levels of expertise are needed to deliver and manage sustainable energy systems, and training on energy efficiency and energy system operation and maintenance are currently not being achieved in humanitarian response. For example, at an institutional level, Grafham and Lahn (2018) found that only 8 out of 21 agencies surveyed as part of a study on humanitarian energy use had a policy for training or advising staff on reducing energy use. At the field level, Fuentes et al. (2018) also found that a lack of training on the operation and maintenance of solar installations led to a dramatic reduction in the lifetime of the systems.

In conclusion, the challenges facing energy provision for humanitarian operations are very different to those associated with accessing energy for displaced and host communities. The logistics and camp coordination sectors have been slow to move to sustainable solutions and risk further damage to the environment, thus exacerbating climate change impacts. Anecdotal evidence also suggests that there is a double standard present in humanitarian responses, namely that more energy resources are available for humanitarians than for displaced people.

## Informal action to comprehensive and integrated energy programming

Humanitarian response has evolved since the establishment of the cluster system and as displacement situations have become increasingly complex and protracted. While there is generally consensus that the cluster system has increased the effectiveness of humanitarian assistance (Humphries 2013), many authors suggest that the response needs to become more comprehensive and sustainable (Mooney 2009; Churruca Muguruza 2015; Agenda for Humanity 2016). In particular, the challenge of cross-sector coordination remains problematic: programming which is not aligned with a single cluster often struggles to work within existing coordination mechanisms, such as energy interventions (Humphries 2013; Sanderson et al. 2015; Sanderson 2017; Knox Clarke and Campbell 2018). Despite some progress, humanitarian organisations, and practitioners are often ill-equipped to meet energy needs (Barbieri et al. 2016; Gerrard 2016; Lehne et al. 2016). We suggest two mechanisms that could help to address this: (1) integrating energy planning with existing humanitarian programming and (2) supporting the humanitarian clusters to take further ownership of energy issues.

### Integrated action on energy: delivering coordinated progress

Integrating sustainable energy into existing humanitarian coordination mechanisms as well as enabling cross-sector collaboration within aid structures is key to meeting global sustainable energy goals and for the humanitarian sector to achieve Sustainable Development Goal (SDG) 7. However, at the global policy level, delivering coordinated progress on energy has been challenging. While several international frameworks have helped to provide an enabling political environment to achieve better integration of energy in humanitarian response, to date, these policy commitments have not translated to a systematic use of sustainable energy solutions in practice. SDG 7 (UNDESA 2019), the Agenda for Humanity (Agenda for Humanity 2016), the New York Declaration for Refugees and Migrants (UN 2016), the Comprehensive Refugee Response Framework (UNHCR 2016), the Global Compact for Safe Orderly and Regular Migration (UN 2018b), and the Global Compact on Refugees (UN 2018a) are key enablers for the integrated action needed for achieving universal energy access in humanitarian contexts. To achieve real change, and despite the challenges present in achieving it, coordinated, multi-stakeholder action is needed to bridge the gap between these commitments and action on the ground.

One of the key priorities identified by humanitarian energy sector practitioners was the issue of how to integrate energy within humanitarian practices and policies at the national and global levels (UNITAR 2018). To help facilitate this, the Global Platform for Actionon Sustainable Energy in Displacement Settings (GPA) was founded in 2018. The vision of the GPA states that “every person affected by conflict or natural disaster has access to affordable, reliable, sustainable, and modern energy services by 2030” (UNITAR 2018). The GPA outlines five working areas on coordination, advocacy, finance, technical expertise and data. Under these GPA working areas, sector practitioners support humanitarian organisations and national refugee-hosting governments to improve the coordination and systematic planning of energy programming, as seen in Rwanda, Bangladesh, Jordan and Uganda (Grafham 2018; Haselip 2018; UNHCR 2019d). Coordination is also undertaken by working with humanitarian clusters to advocate for improved action on energy, by evaluating gaps in expertise and resources, and by supporting agencies to take ownership of the energy issues faced in displacement settings. We have identified three initial areas where humanitarian organisations and GPA partners can work together to further integrate action on energy.

First, evidence suggests there is a severe shortage of energy expertise in the humanitarian system and no systematic approach to planning for and managing energy provision (Lahn and Grafham 2015; Barbieri 2019). To help bridge this expertise gap, the GPA is working to support knowledge transfer from the development and private sector to the humanitarian context through expert secondments (NORCAP 2019). Training programmes under development aim to equip humanitarian practitioners with the skills needed to better provide sustainable energy interventions for displacement contexts. Humanitarian organisations and partners can become directly involved in shaping this transition by joining the GPA workstream on technical capacity or attending technical trainings such as those led by Mercy Corps and Energypedia (Mercy Corps 2019; Energypedia 2020d).

Second, understanding the need for energy in humanitarian settings needs to be underpinned by high-quality and usable data (Haselip 2019). However, data is not routinely collected on energy and no standardised methods exist for measurement and reporting (Corbyn and Vianello 2018). Partners following the GPA working area on data and evidence are working to assess whether energy indicators can be standardised and to support the development of an energy assessment toolkit. This work draws on established frameworks such as the Multi-Tier Framework (ESMAP 2015) to embed learning from the development sector into humanitarian programming, in a similar way to how the cash assistance, inclusion and gender sectors integrated learning within existing aid processes (Haselip 2019).

Third, innovative ways of working and private sector investment could have a considerable role in developing energy solutions in displacement settings. Recent research suggests that creating and supporting dynamic markets for energy requires long-term planning and investment, and that humanitarian systems need to be supported by private energy investors and suppliers to deliver sustainable energy access for their humanitarian facilities through outsourcing the management of the energy and purchasing it as a service rather than procuring and operating diesel-based infrastructure (Bisaga and Huber 2020). The humanitarian energy sector is gradually moving towards this goal, guided by the partners involved in the GPA working area on innovative financing and decarbonising humanitarian energy (Gibson 2020).

Further work is required to ensure that the changes being explored through these working areas are embedded within humanitarian programming and that sustainable energy solutions can be realised by humanitarian clusters. The section below outlines some suggested practical recommendations that might be possible and the types of activities that individual organisations and humanitarian programmes could adopt to ensure that energy access for all displaced people starts to become a reality.

### Supporting cluster action on energy: recommendations

The need for a comprehensive response on energy is increasingly pressing. However, instead of advocating for a separate energy cluster, our approach recommends integrating energy into the existing clusters and working through existing processes such as those developed under the GPA working areas. To support this integration and to ensure the provision of energy in humanitarian situations, we have developed ten recommendations for the humanitarian sector:

1. Advocate for access to energy for displaced people and host communities: commit to sustainable energy objectives in internal and external policy and public documents.
2. Develop existing technical capacity and sustainable energy implementation plans within organisations that are led by dedicated energy specialists within cluster teams.
3. Work with GPA partners to develop a roadmap on embedding energy within each cluster, specifically outlining sector-specific energy needs and solutions.^2^
4. Develop sustainable energy recommendations that target specific areas of the humanitarian response system such as donors, policy makers, cluster leads and humanitarian agencies.^3^
5. Measure the overall progress on energy access levels using standard indicators currently being developed by the GPA:^4^
  - Each cluster should lead specific energy indicators related to its remit.
  - Ensure energy expenditure is captured in minimum expenditure baskets and ensure sustainable energy products are included within the supply of household items.
6. Aim for Tier 3 standards (ESMAP 2015) for household electricity access and provide access to electricity in public spaces and Tier 4 standards (ESMAP 2015) on indoor air pollution for improved cookstoves and support long-term cooking solutions.
7. Adopt Lighting GLobal (2018) standards for standalone products to ensure quality and safety.
8. Integrate cooking fuel needs into cash and voucher programming and learn from new innovative approaches to supply cooking energy in humanitarian settings (Vianello 2016).
9. Transition away from freely distributed products in all but emergency situations and work with local and private sector partners, livelihoods, cash and GBV specialists, on strategies for the sustainable delivery of energy solutions (Whitehouse 2019; Bisaga and Huber 2020).
10. Ensure that programming is inclusive and engages displaced people directly in programme design and delivery processes: where possible employ displaced people and develop livelihood components of energy programming (Rosenberg-Jansen 2020).

The following sections provide recommendations on which energy-related issues the existing clusters can take ownership of in order to ensure a more coordinated and comprehensive response.

#### Protection cluster: advocate to ensure energy needs are sufficiently met

The protection cluster could take the lead on analysing the risks associated with a lack of energy access. It could also aim to play a larger role in advocating for energy provision based on the protection risks that access to energy can help mitigate. Remits could be expanded beyond gender-based violence, which has been a major focus to date, to include the risks of refoulement, the impacts of a lack of energy resources on wellbeing, problems associated with environmental protection, and engagements on energy with local host communities. The protection cluster could work to develop guidance on inclusion and protection principles for energy and provide guidance on energy as a tool to mitigate gender-based violence. This approach would recognise the protection remit while also being progressive in supporting displaced people to become self-sufficient. It may also enable the protection cluster to work more closely with other sectors, such as the shelter and WASH clusters, to deliver multi-sectoral approaches.

#### Shelter cluster: meet household electricity needs

The remit of the shelter cluster already encompasses some elements of household electricity needs. However, the cluster needs to integrate sustainable energy products into NFIs and ensure that any products provided meet the needs of displaced communities. Reporting on the energy needs of households as has been done in Bangladesh and Venezuela should also become standard practice. The cluster could also work with organisations such as Sphere to ensure energy is more clearly recognised with the Sphere standards. In the longer term, the cluster could try to move beyond free distribution models in all but emergency situations by considering how energy services can also be provided via market-based delivery models. The shelter cluster could also focus on household electricity needs beyond lighting and mobile phone charging to support a wider range of energy needs. While recognising immediate shelter needs in emergency contexts, the cluster should aim to integrate energy efficiency and support vernacular shelter design that uses local materials (taking into account local supply capacity and possible market distortions) whenever possible.

#### Food security and nutrition: enable access to household cooking services

The food security and nutrition clusters should adopt more responsibility for providing household cooking services, by working with the shelter/NFI cluster. The distribution of cookstoves to displaced communities has had limited success, therefore the clusters could try to work with initiatives such as the Modern Energy Cooking Services programme (Loughborough University 2020), the Clean Cooking Alliance (Clean Cooking Alliance 2020), and the private sector to trial alternative solutions. The cluster could also attempt to implement guidance developed by the Power Agriculture programme to support food production, processing and consumption (Energypedia 2020c).

#### Health, WASH and education: use sustainable energy to power community services

The health, WASH and education clusters could lead the provision of energy within their facilities. Progress towards this has already been achieved in some areas such as powering health clinics and implementing solar and hybrid water-pumping systems. These clusters could also utilise their existing expertise to take the lead on attempts to provide energy for community facilities such as public street lighting. This could be achieved by working with health and energy specialists and existing resources, such as those produced by SE4ALL (2020), (Llario and Kiprono 2020), and Energypedia (2020d).

#### Camp coordination and logistics: decarbonise energy infrastructure and lead by example

The camp coordination and logistics clusters could assume more responsibility for delivering sustainable energy for humanitarian operations which is likely to result in substantial cost savings. The CCCM cluster could also take responsibility for overall support on energy issues and ensure that the voices of displaced people are represented in decision-making processes. This could be supported by facilitating involvement in cluster coordination meetings and processes such as the Joint Intersectoral Analysis Framework. The cluster should also build on existing work to purchase electricity as a service (Gibson 2020) and support attempts being made to adopt long-term contracts which can enable the provision of sustainable and lower cost electricity over time.

## Conclusion: delivering progress on sustainable energy access

The importance of providing energy in humanitarian settings is too often overlooked or inadequately prioritised. However, its exclusion from the cluster system does not mean that action cannot be taken. Existing commitments and movement on energy are already being demonstrated by the number of energy initiatives already undertaken in the humanitarian landscape. Progress towards improving energy provision is also demonstrated by the inclusion of displaced people in key energy policies and initiatives.

There is an increasing recognition amongst practitioners of the role energy services play in underpinning many displaced communities' needs and the help it provides in supporting the objectives of the humanitarian clusters, particularly food and nutrition, health, protection, WASH and education. However, our analysis demonstrates that humanitarian clusters should carefully consider energy to enhance the well-being and protection of displaced populations and to improve the sustainability of operations.

There has been a substantial evolution of the energy and humanitarian response sectors during recent years. Change has been enabled by the international community’s commitments to new climate, sustainability and humanity agendas that push for a change in the status quo, and progression at the field and local levels. The affordability and accessibility of sustainable energy solutions provides further reasons to be optimistic. The question now is, how can the energy agenda in forced displacement settings be owned and scaled? Success will likely depend on continued commitment at both programme and policy levels. The work of the GPA can support this, however, it is vital that the link between energy and the clusters are more explicitly recognised and acted upon by donors, implementing agencies and coordinating bodies to progress the sector further.

Overall, our findings highlight the inherent value of energy in all our lives. It is the electricity we use to power our homes, it is the fuel we use to cook our food and heat or cool our houses, and it underpins how and where we travel. Without it we cannot access the internet, do our jobs or hobbies, and our quality of life starts to rapidly diminish. Access to modern energy has widely been accepted as an essential component of Western lives, as well as being critical for communities to feel safe and supported. The same is often not true of the lives of refugees, IDPs and the local host community. Now is the time for this to change. This paper has presented recommendations for the humanitarian sector to embed energy across the clusters and within existing ways of working. Delivering these reforms need not be a difficult or expensive exercise: private sector and development partners are waiting and ready to support these changes; however, champions from within the humanitarian sector are needed to step forward to support this transition. If achieved, the improved quality of life enabled by access to energy can be a bridge to cohesion and development, as sustainable energy is transformative in the recovery of crisis-affected communities.

## Data Availability

Data sharing is not applicable to this article as no datasets were generated or analysed.

## Abbreviations

CCCM: Camp Coordination and Camp Management
FAO: Food and Agriculture Organization
GPA: Global Platform for Actionon Sustainable Energy in Displacement Settings
IASC: Inter-agency Standing Committee
IDP: Internally Displaced People
IOM: International Organization for Migration
LPG: Liquefied petroleum gas
MEI: Moving Energy Initiative
NFI: Non-food items
NGO: Non-governmental organisation
OCHA: United Nations Office for the Coordination of Humanitarian Affairs
RCM: Refugee Coordination Model
SGBV: Sexual and Gender-based violence
SDG: Sustainable Development Goal
UNHCR: United Nations High Commissioner for Refugees
UNICEF: United Nations Children’s Fund
WASH: Water, sanitation and hygiene
WFP: World Food Programme
WHO: World Health Organization

## Footnotes

1 This is the duration of a system-wide response to a sudden onset or rapidly deteriorating humanitarian situation under the IASC system (UNHCR 2015).

2 Please contact Aimee Jenks (aimee.jenks@unitar.org) for more information.

3 For different topic areas please reach-out to the working group chairs of the GPA. Online here.

4 Please contact Iwona Bisaga (i.bisaga@ucl.ac.uk) or Sarah Rosenberg-Jansen (Sarah.Rosenberg-Jansen@qeh.ox.ac.uk) on this topic.
